# Analysis of laser Doppler flowmetry long-term recordings for investigation of cerebral microcirculation during neurointensive care

**DOI:** 10.3389/fnins.2022.1030805

**Published:** 2022-11-03

**Authors:** Stina Mauritzon, Fredrik Ginstman, Jan Hillman, Karin Wårdell

**Affiliations:** ^1^Neuroengineering Lab, Department of Biomedical Engineering, Linköping University, Linköping, Sweden; ^2^Department of Neurosurgery and Department of Biomedical and Clinical Sciences, Linköping University, Linköping, Sweden

**Keywords:** human brain, laser Doppler flowmetry (LDF), neurointensive care unit (NICU), microcirculation, vasomotion, cortical spreading depolarizations (CSD), signal analysis

## Abstract

Cerebral blood flow is monitored in the neurointensive care unit (NICU) to avoid further brain damage caused by secondary insults following subarachnoid hemorrhage and brain trauma. Current techniques are mainly snap-shot based and focus on larger vessels. However, continuous monitoring of the smaller vessels may help detect the onset of secondary insults at an earlier stage. In this study, long-term measurements of brain microcirculation with laser Doppler flowmetry (LDF) were performed and evaluated. The aim was to identify and describe physiological signal variations and separate these from movement artifacts. Fiberoptic probes for subcortical LDF recordings of perfusion and total light intensity (TLI) were implanted in three patients with subarachnoid hemorrhage. Data were successfully collected and visualized in real-time over 4 days, resulting in 34, 12, and 8.5 h per patient. Visual observation, wavelet transforms, moving medians, and peak envelopes were used to identify and describe movement artifacts and physiological changes. Artifacts occurred in <5% of the total recording time and could be identified through signal processing. Identified physiological signal patterns included a slowly increasing perfusion trend over hours, vasomotion mainly at 2 cycles/min both in the perfusion and the TLI, and rapid, synchronized changes in the TLI and the perfusion on 38 occasions. Continuous LDF recordings indicating changes in the microvascular blood flow can increase the understanding of the microcirculation in the injured brain. In the long run, this may become a complement for the detection of secondary insults at an earlier stage than possible with today’s techniques.

## Introduction

In the neurointensive care unit (NICU), patients with subarachnoid hemorrhage (SAH) or traumatic brain injury (TBI) are monitored to avoid further brain damage. Especially in subarachnoid hemorrhages, the primary injury initiates pathophysiological events that, if left untreated, may lead to delayed cerebral ischemia (DCI) or even infarction. These complications usually appear without clear warning signs 4–14 days after the injury (Neifert et al., 2021).

The complex physiology requires monitoring of cerebral blood flow (CBF) and other physiological parameters to detect such harmful changes in time for treatment. One common cause of DCI is vasospasm, but recent research suggests multiple reasons (Geraghty and Testai, 2017), such as impaired cerebral autoregulation (CA), which refers to the brain’s ability to keep CBF continuous despite changes in cerebral perfusion pressure (CPP) (Fantini et al., 2016), and cortical spreading depolarizations (CSD) (Leao, 1944). CSD is a prolonged depolarization wave in nerve cells propagating with a few millimeters per second in gray brain matter (Woitzik et al., 2013). Similar excitation waves have been observed in the deeper parts of the brain during stereotactic neurosurgery (Sramka et al., 1977). In healthy tissue, CSD is followed by local reactive hyperemia due to the increased neural activity and energy demand (Hoffmann and Ayata, 2013; Dreier et al., 2017; Geraghty and Testai, 2017), while an inverse hemodynamic response often is seen in the injured brain. A third important mechanism in the regulation of local blood flow is vasomotion (Intaglietta, 1990; Aalkjær et al., 2011; Cole et al., 2019), which is a slow, rhythmic variation of the microvessel diameter, resulting in similar rhythmic oscillations of the microvascular blood flow. Despite being studied by many researchers, the exact mechanism behind vasomotion is still not clarified (Aalkjær et al., 2011).

Current methods relating to CBF measurements include for example transcranial Doppler ultrasound (TCD), thermal diffusion monitoring, and magnetic resonance imaging (MRI), where the former focus on large vessels. Both TCD and MRI, however, only give snap-shot overviews of cerebral perfusion. Real-time monitoring of the microvascular blood flow in the NICU is a challenging task but can give additional information about the brain’s status. Several research groups have presented methods for human CBF measurements and approaches for the investigation of the CA (Tahhan et al., 2021). Promising results have been shown with different optical methods, such as spectroscopy (Kim et al., 2010; Rejmstad et al., 2016a; Seule et al., 2016; Busch et al., 2018) and laser Doppler flowmetry (LDF) (Kirkpatrick et al., 1994; Rejmstad et al., 2018).

Laser Doppler flowmetry (Nilsson et al., 2003) is a technique widely used for studying microcirculation in cutaneous tissue but has only been applied in a few investigations of human cerebral tissue. Examples from our group are studies about deep brain stimulation (DBS) implantation (Wårdell et al., 2013; Zsigmond et al., 2017), brain tumor surgery (Rejmstad et al., 2016b; Richter et al., 2021), and NICU monitoring (Rejmstad et al., 2018). For brain tissue measurements in the NICU, LDF has been modified for simultaneous recording of microvascular blood flow (also denoted perfusion) and total light intensity (TLI); the latter allowing for variations in gray-white tissue intensity to be observed. The perfusion and TLI signals have been evaluated during DBS surgery in close to 3,000 well-defined anatomical brain tissue measurement positions (Zsigmond et al., 2017). LDF has also been combined with electrocorticography (ECoG) for the measurement of CSDs by using optodes integrated into the ECoG recording strips placed on the cortex (Dreier et al., 2009). As detection of CSDs with ECoG requires direct contact with the cortex, it has limitations in routine NICU use since multiple monitoring devices are required. Post-operative and intra-operative alternatives for CSD detection are presented in Woitzik et al. (2013), including for example changes in cortical reflectance measured by intrinsic optical signal (IOS) imaging.

To bring LDF one step closer to clinical use in the NICU, several aspects need to be considered, including the sensitivity to movement artifacts. Post-processing of LDF inherited signals such as heart rate (HR), breathing, and slow and fast vasomotion have previously been presented (Bernjak and Stefanovska, 2007; Stefanovska, 2007). Peak-to-peak (P-P) of the perfusion, pulsative index, and trend presentations based on different pre-set intervals have also been introduced (Rejmstad et al., 2018). However, there is still a need to develop methods for real-time detection of signal components relevant to neurointensive care, that can give feedback on both trends as well as relevant changes on the minute and second scale. Ideally, different physiological events recorded with LDF should be possible to separate from movement artifacts.

This study aimed to record and analyze long-term LDF brain tissue signals obtained from patients in the NICU. The analysis included identification and post-processing of movement artifacts and physiological patterns such as trends, vasomotion, and other physiological changes in the LDF data that can be relevant for neurointensive care.

## Materials and methods

**FIGURE 1:**
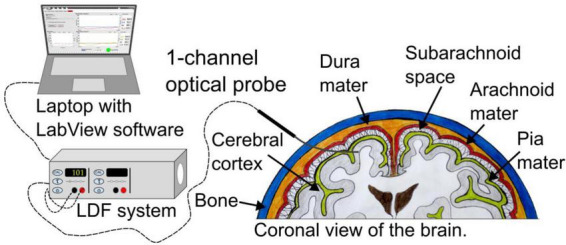
Overview of the system setup. The probe is placed in the subcortical brain matter.

An LDF system (PF 5010, Perimed AB, Sweden) with a bandwidth of 20 to 15 kHz previously adapted for neurointensive care was used. The LDF system communicates with an in-house developed software designed in LabVIEW (National Instruments Inc., Austin, TX, USA) for real-time acquisition (*f*_*s*_ = 100 Hz) and presentation of the signals. A flexible optical probe (∅ = 1.7 mm) with a catheter-like design adapted for brain tissue measurements was implanted in the subcortical brain matter (Rejmstad et al., 2018). For an overview of the setup, see Figure 1.

A thin stainless-steel wire in the center of the probe tip served as a marker during investigations with computed tomography (CT). Low power laser light (≤2 mW, λ = 780 nm) was transmitted to the tissue through the probe and led back to the detector after interaction with the tissue for processing of perfusion (i.e., microvascular blood flow) and TLI. The perfusion and TLI are presented in arbitrary units [a.u.] ranging from 0 to 999 and 0 to 10, respectively. Before and after the inclusion of a new patient, the performance of the probe and the LDF system was tested using a standard microsphere solution (PF 1001 Motility, Perimed AB, Sweden). If necessary, the system was recalibrated. The probe was carefully cleaned and sterilized according to the Sterrad^®^ protocol (Jacobs, 2006).

### Patients, probe insertion, and measurements

Laser Doppler flowmetry data from three SAH patients (age 43, 45, and 69, two males and one female) enrolled in the NICU were used for the analysis. Data from one patient has been published in Rejmstad et al. (2018). The measurements were approved by the local ethics committee (EPN 2018/322-31, 2021-03527). As the patients were unconscious upon arrival in the hospital, informed consent was obtained from the next-of-kin before inclusion.

The flexible tip of the LDF probe was manually inserted 1–2 cm into the subcortical gray-white matter transition zone during the same neurosurgical procedure as the implantation of an intracranial pressure (ICP) device and bilateral microdialysis catheters (M Dialysis AB, Sweden). The fiber-optical probe was secured by a fixation wing sutured on the skin surface next to the entry of the probe to reduce external movement artifacts. The TLI signal, indicating the change in tissue brightness, was used during insertion to verify the position. When assuming subcortical gray-white matter, the perfusion signal was used to assure sufficient microvascular blood flow in the measurement volume. After probe fixation, a test measurement was done to ascertain signal quality before the patient was transferred to the NICU. A second test measurement was performed upon arrival in the NICU. According to the clinic’s routine, the positions of the catheters and the optical probe were verified by a conventional bedside CT (CereTom, NeuroLogica Corp., Danvers, MA, USA). Patients were followed by serial CT-scans as per clinical routine and there were no signs of clinical or radiologic adverse effects directly attributable to the LDF probe.

The test recordings were followed by one or two measurement sessions per day lasting at least one hour. Data were continuously stored and presented in real-time together with trend curves based on averaging over 20 s intervals. The LDF time constant (τ) was set to 0.03. Careful notes were taken to link the possible influence of drugs or external movement artifacts such as head or cable movements and medical care to the LDF signals. Notes of HR, mean arterial pressure (MAP), and ICP were taken from the standard patient monitoring system. In addition to ICP and microdialysis, continuous EEG (cEEG) was part of the monitoring equipment.

### Signal analysis

The 12 h of recordings from Patient 2 were used for the initial analysis of the signals. The analyses of the observed patterns were then carried out on all data.

#### Signal components

The perfusion signal includes different frequency components caused by cardiovascular and respiratory fluctuations (Stefanovska, 2007). Vasomotion occurs at 0.005–0.145 Hz, respiratory rate at 0.145–0.6 Hz, and HR at 0.6–2.5 Hz. The vasomotion range can be further split into shorter intervals depending on the vasomotor origin (Stefanovska, 2007), which has been investigated based on animal and human skin measurements with LDF. Pressure changes, metabolic need, or pathological mechanisms affecting the microvascular blood flow will cause variations in the perfusion signal over time. Patterns of interest for this study include perfusion and TLI changes observed over hours, minutes, and seconds.

#### Analysis tools

To analyze the time and frequency content in the data, the Signal Processing and the Wavelet Toolboxes in MatLab (ver. R2018b, The MathWorks Inc., Natick, MA, USA) were used.

For time-frequency analysis of the perfusion, which requires good resolution both in time and frequency, the continuous wavelet transform (CWT) was utilized for its benefit of varying window lengths for different frequencies. The analytic Morlet was chosen as the mother wavelet.

The power spectral density (PSD), Y[k], of a signal segment k, was estimated according to


(1) $$Y\;\left[k\right]=\frac{1}{N}\,{\left|X\,\left[k\right]\right|}^{2},$$

where *N* is the length of the window and *X*[*k*] the Fourier transform (FT) of that segment. To compute the FT of the signal, the Fast Fourier Transform algorithm was used. The PSD was calculated for comparison with the wavelet transform (WT).

To analyze the variation in amplitude of the perfusion signal, P-P values were calculated as the upper peak envelope minus the lower peak envelope. The level of the perfusion signal was calculated using a moving median to avoid influence from movement artifacts.

#### Workflow for signal analysis

**FIGURE 2:**
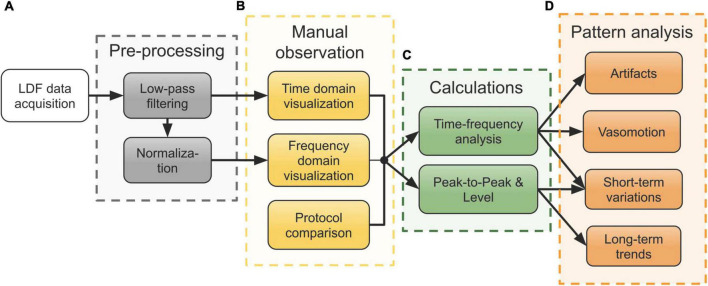
Workflow of the processing steps of data acquired in the neurointensive care unit (NICU). **(A)** Pre-processing of the raw laser Doppler flowmetry (LDF) data. **(B)** Manual observation of the signals in both the time and frequency domain, compared with the measurement protocol notes. **(C)** Time-frequency analysis using wavelets and computation of peak-to-peak (P-P) and level of the perfusion signal for further analysis of patterns. **(D)** Analysis of various patterns seen during the manual observation using the results from panel **(C)**.

The overall processing steps after data acquisition are shown in Figure 2. The data was first low-pass filtered (3rd order Butterworth) with a cut-off frequency of 10 Hz to filter out possible unwanted high-frequency components. Before frequency domain visualization, the perfusion signal was normalized by subtraction and division with its mean (obtained by low-pass filtering, 3rd order Butterworth, cut-off frequency 0.005 Hz).

As an initial step after pre-processing (Figure 2A), both time and frequency domain plots were used for comparison with the measurement protocol. The measurement protocol contains notes on external factors and medical treatment possibly influencing the signals. This step is marked in Figure 2B and served as a means to identify interesting physiological patterns and movement artifacts for further analysis.

Patterns observed in this initial step included fluctuating level and P-P values of the perfusion as well as varying spectral content both in the perfusion and the TLI. Figure 2C summarizes the calculations that were done to describe these patterns in terms of signal components. For time-frequency analyses, the WT was calculated using CWT. The level of the perfusion was calculated as a moving median over 5-second intervals and P-P values were calculated based on peak envelopes interpolated over 50 samples. No normalization of the perfusion signal was done before calculating the level and P-P values.

As a final step, a more detailed analysis based on these quantities was done for each of the four observed patterns presented in Figure 2D: movement artifacts, vasomotion, short-term variations, and long-term trends. The arrows from the boxes in Figure 2C show which quantities were used for each analysis.

#### Movement artifacts

Spectral analysis revealed a broader spectrum of high amplitude frequencies for external movement artifacts noted in the measurement protocol, as compared to non-artifact areas solely consisting of the expected frequencies.

The CWT was used to obtain a time-scale representation of the perfusion signal. By integrating the square of the WT over the scales corresponding to 0.5–0.6 Hz for each time sample, a time-resolved curve of the wavelet energy was obtained. The energy in this frequency band is close to zero for stable data and higher for artifacts. A threshold of 0.01, based on visual observation in the initial analysis, separated artifacts from physiological data.

To evaluate the choice of threshold, it was tested on all data from all three patients. The result was then compared to notes in the measurement protocol and visual observation of the perfusion signal in the time domain. The number of artifacts was presented as the mean (m) ± standard deviation (SD).

#### Vasomotion

Further analysis of vasomotion patterns included identification of the frequency components. The 1D-integral of the squared magnitude of the WT over time resulted in frequency plots for shorter segments (approximately 10 min) of all data sequences. The vasomotion peaks, if any, were noted for each segment.

#### Short-term variations

In the initial manual observation of signal patterns, a repetitive short-term increase in the TLI accompanied by a decrease in the perfusion was seen that had no external cause according to the protocol. In some occurrences, a short perfusion peak followed the recovery of the signal.

To analyze this pattern further, the TLI signal was low-pass filtered (3rd order Butterworth, cut-off 0.5 Hz) to smooth rapid oscillations related to the HR. The duration, amplitude, and time between occurrences were then calculated.

In the perfusion signal, level and P-P decrease was calculated for each occurrence, as well as the duration of the decrease. The number of perfusion peaks following the recovery was noted as well.

Spectral content, based on the time-frequency analysis, was examined for each occurrence.

#### Long-term trends

A slowly increasing trend in the perfusion and stable TLI was found during the manual observation in the time domain. To investigate the change over time in the perfusion signal, the mean of the level and P-P values for 5-min intervals were calculated and compared. To see the variation between two adjacent intervals, the percentage increase (or decrease) and the ratio between the two measures were calculated.

## Results

**FIGURE 3:**
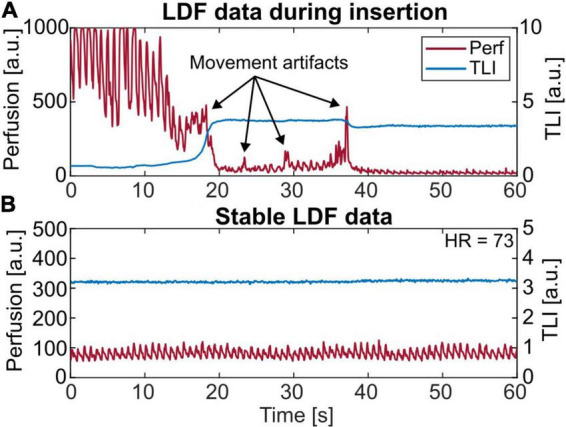
**(A)** A 60 s laser Doppler flowmetry (LDF) recording showing the insertion of the probe. About 18 s into the recording the total light intensity (TLI) increases rapidly since reaching brighter tissue. During insertion, movement artifacts are seen in the perfusion signal, marked with arrows. **(B)** Short-term (60 s) LDF data presenting stable microvascular blood flow. Note the range differences on the axes.

All three optical probes were successfully implanted in the subcortical gray-white matter. In one case, the probe stopped functioning due to involuntary movements of the head when the patient was waking up on day 4. During insertion, the TLI signal indicated passage through gray matter toward white tissue and could thus be used as information to find a suitable position. An example is seen in Figure 3A, where the TLI goes from approximately 1 to 4 a.u. in 2 s. While slowly moving the probe into the brain, artifacts in the perfusion signal were generated. These are marked by arrows in the figure. A short sequence of stable LDF data is shown in Figure 3B.

In total, 34, 12, and 8.5 h of LDF data were recorded in the NICU during 3–4 days, mainly in 2-h sequences on all three patients. The calculated HR from the perfusion signal agreed with the monitored HR.

### Movement artifacts

**FIGURE 4:**
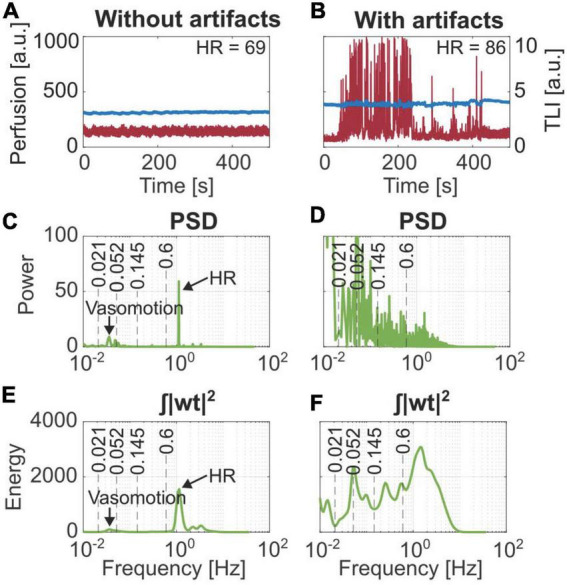
Comparison of the frequency content based on Fourier transform (PSD) and Wavelet transform (| wt| ^2^). **(A)** Stable laser Doppler flowmetry (LDF) data. **(B)** Data containing movement artifacts. Frequency components for vasomotion and heart rate (HR) are marked in panels **(C,E)** from the stable data, which cannot be distinguished in panels **(D,F)** belonging to the artifact region.

Examples of recordings over 500 s without and with movement artifacts are presented in Figures 4A,B. Their corresponding PSD and wavelet energy are presented in Figures 4C–F. Movement artifacts could be distinguished using the notes from the measurement protocol. Typical artifact spikes in the time domain perfusion signal corresponded with high amplitude, noisy frequency spectra where the expected frequencies could not be distinguished in the PSD. Similar effects were seen in the energy plots of the WT.

On average, 4.7 ± 4.4% of all data points were identified as artifacts based on the spectral content. This is compared to artifacts visually observed in the time domain and artifacts noted in the measurement protocol, summarized in Table 1.

**TABLE 1:** Result from the identification of artifacts based on spectral content, presented as mean ± standard deviation, compared to visual observations in the time domain and artifacts as noted in the measurement protocol.

Patient	Protocol (%)	Visual (%)	Identified (%)
1	–	5.5 ± 5.9	6.5 ± 5.0
2	10.6 ± 14.0	7.0 ± 9.5	5.5 ± 6.6
3	7.5 ± 7.9	3.2 ± 2.3	2.1 ± 1.7
Average	9.1 ± 10.9	5.3 ± 5.9	4.7 ± 4.4

Artifacts noted in the protocol are given as the range of, for example, one nursing session, which may include moments of no artifacts in between.

**FIGURE 5:**
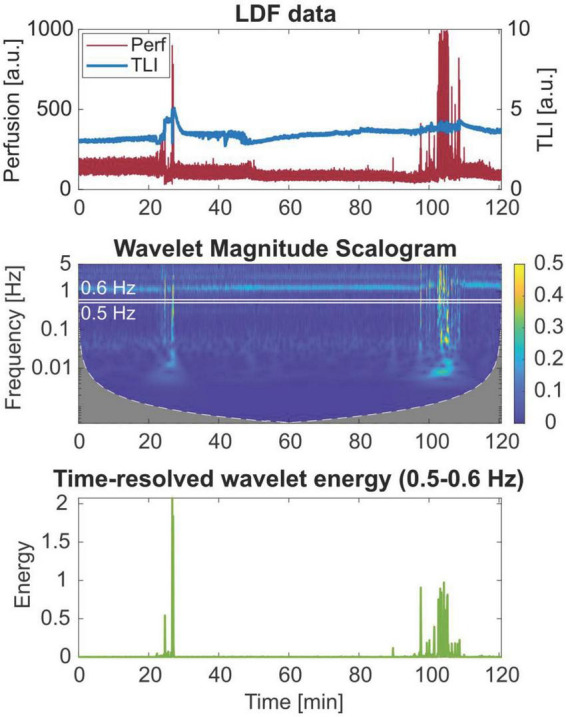
Wavelet scalogram of the perfusion signal and time-resolved wavelet energy in the 0.5–0.6 Hz frequency band of a 2-h laser Doppler flowmetry (LDF) time series, showing broadband artifact data in the frequency domain.

Figure 5 shows the perfusion signal in the time domain, the corresponding magnitude scalogram of the WT, and the time-resolved wavelet energy in the 0.5–0.6 Hz frequency band. The scalogram presents the magnitude of the WT in the time-frequency plane.

**FIGURE 6:**
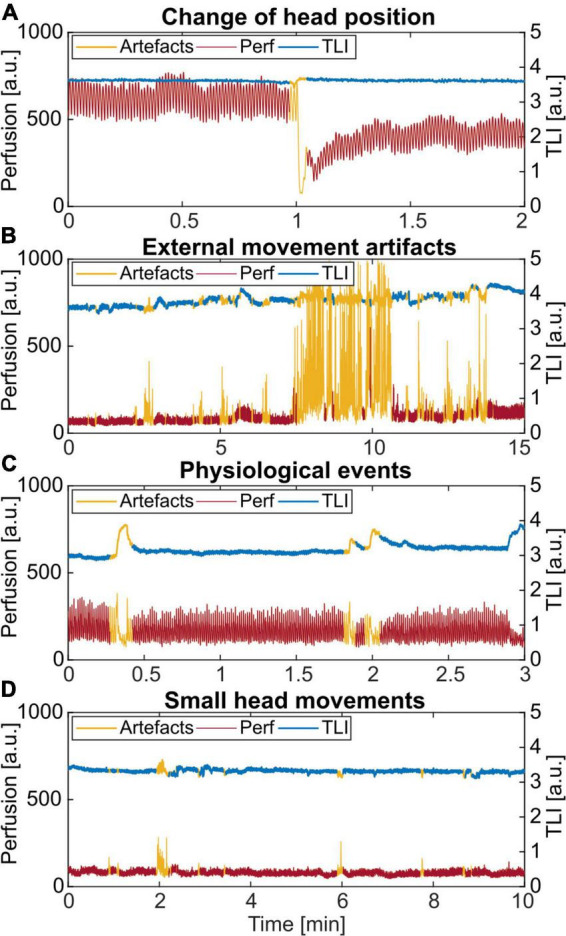
Examples of different artifacts identified by thresholding the time-resolved wavelet energy in the 0.5–0.6 Hz frequency band. **(A)** Change of the head position. **(B)** External movement from nursing and care of the patient. **(C)** An unknown physiological pattern. **(D)** Small spikes caused by involuntary head movements by the patient. Only the artifact at minute 2 was noted in the protocol.

Four examples of artifacts identified by thresholding the time-resolved wavelet energy are shown in Figure 6. In Figure 6A, the head position was slightly moved when EEG electrodes were changed, resulting in a drop in the perfusion. Typical spikes caused by external movements, adding to the perfusion value, are seen in Figure 6B. In Figure 6C, the identified areas are not due to an external movement. However, the frequency analysis showed the typical broadband spectrum belonging to artifact data. Finally, in Figure 6D, small spikes caused by involuntary movements of the head by the patient were identified. In this case, only the slightly bigger artifact at minute 2 was noted in the protocol.

### Vasomotion

**FIGURE 7:**
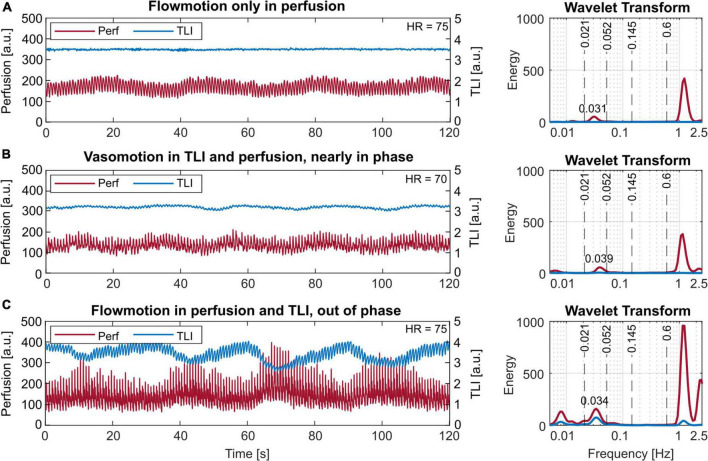
Short-term (120 s) laser Doppler flowmetry (LDF) data with different vasomotion patterns in the time (left) and frequency (right) domain. **(A)** Vasomotion only in the perfusion signal. **(B)** Vasomotion in the perfusion and slightly also the total light intensity (TLI), nearly in phase. **(C)** Vasomotion both in the perfusion and the TLI, 180 degrees out of phase. The observed rates correspond to 1.86 cycles/min or 0.031 Hz in panel **(A)**, 2.34 cycles/min or 0.039 Hz in panel **(B)**, and 2.04 cycles/min or 0.034 Hz in panel **(C)**. Note the log-scale of the *x*-axes, used for better visibility in the frequency plots.

Three examples of cyclic signal variations are presented in Figure 7. Vasomotion is seen in the perfusion signal in Figure 7A and both in the perfusion and TLI signals in Figures 7B,C. The oscillations were nearly in-phase in Figure 7B while phase-shifted 180 degrees in Figure 7C. In these examples, the rate of oscillation was approximately 2 cycles/min.

In all data, rates of 0.9–3 cycles/min (0.015–0.05 Hz) were found, with the most common frequency being 2 cycles/min (0.033 Hz).

### Short-term variations

**FIGURE 8:**
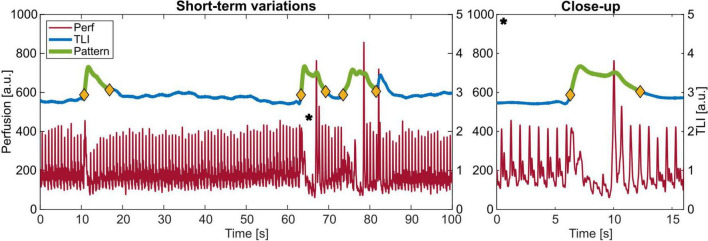
Example of the observed pattern with increasing total light intensity (TLI) and a decrease in perfusion with a close-up to the right of *.

Three examples of the observed short-term signal patterns are given in Figure 8, with a close-up of one denoted with a * to the right. Some had a succeeding perfusion spike when the blood flow returned to the level before the event, and some did not. In the frequency domain, this pattern is similar to a movement artifact spectrum (see Figure 6C).

The total number of events, duration, time in-between clustered events, and TLI and perfusion change are summarized in Table 2.

**TABLE 2:** Characteristics of the short-term variations observed in the measurement data from Patient 2.

Patient	Total no.	Time between clustered events (s)	Duration (s)	TLI increase (a.u.)	P-P_perf_ decrease (%)	Spike amplitude (a.u.)
2	38 (11)	59 (9–311)	6 (3–14)	0.17 (0.02–0.89)	51 (20–77)	767 (259–900)

Duration, TLI increase, P-P _Perf_ decrease, and succeeding spike amplitude are given as median (min-max) values. The total number of observed events with a succeeding spike is given in parenthesis after the total number of observed events.

### Long-term trends

**FIGURE 9:**
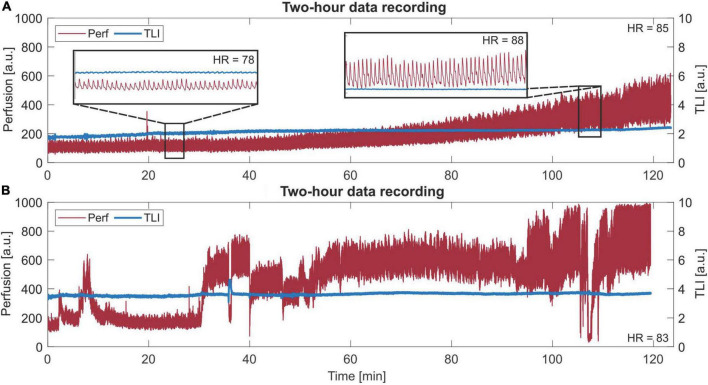
Long-term (120 min) laser Doppler flowmetry (LDF) data. **(A)** A slowly increasing trend in the perfusion signal. Two zoom-in areas of 30 s are given, where a different morphology of each beat in the perfusion signal can be seen for each respective area. The given heart rate (HR) is the mean HR of the whole sequence. Observe the difference in HR between the two zoom-ins. **(B)** Data with more dynamic changes in levels and peak-to-peak values.

Two long-term measurement sequences are presented in Figure 9, revealing the slowly increasing trend in the perfusion signal in Figure 9A and varying levels and P-P values in Figure 9B.

In Figure 9A, the trend increases both in perfusion level and P-P at approximately the same rate, which was revealed after comparing the increase in these two parameters for all data from Patient 2. For each 5 min interval, the level and P-P increased by 5–15%, compared to the whole range of 0–168% increase. In Figure 9B, a 2-h sequence showing dynamic changes in levels and P-P values is presented.

## Discussion

In this study, long-term brain microcirculation signals from three SAH patients were included to investigate the suitability and relevance of LDF in the NICU. Stable, long-term measurements with LDF in the NICU are possible, revealing a dynamically changing microvascular blood flow relating to the physiology of the brain. Different physiological patterns were visible in the time domain. Despite the sensitivity to movement artifacts, the stability in the data collection makes LDF suitable for this patient group, but a small number of remaining artifacts need to be handled.

### Movement artifacts

External movement artifacts are a well-known problem in LDF as the signal relies on the interaction between the laser light and the movement of the red blood cells in the tissue (Nilsson et al., 2003). Any other movement will contribute to the perfusion causing an overestimation and it is therefore of utmost importance that the probe and the surrounding tissue are kept as still as possible during recordings. For cerebral tissue, this sensitivity in the perfusion signal becomes obvious during the surgical implantation of the probe toward the fixation site (Figure 3A). Once fixated, the signal stabilizes, and the typical heart-beat pulsations appear. The small amount of movement artifacts during neurointensive care suggests that LDF is a suitable tool for NICU patients. Despite this, movement artifacts can appear, mainly during nursing and care of the patient. To be useful in clinical practice without interaction being required from the clinical staff, these artifacts need to be handled in real-time by the system. One way to handle this is by looking at the spectral content as described in Section “Movement artifacts.”

Based on the spectral content, 4.7% of the data was identified as artifacts, compared to 5.3% during manual inspection of the time domain data. Several reasons may explain this difference. First, by visual inspection of the time domain data, small sections in between perfusion spikes were included in the calculation. Second, thresholding the wavelet energy by a fixed value requires noise-free data to work perfectly. The perfusion signal varies in a wide range both in level and peak-to-peak, where higher pulsations introduce more noise in the data. This difference is, however, not essential from a clinical perspective but there are things to consider from a signal processing perspective that may be relevant if to be used in real-time.

Another thing to consider is that there seem to be physiological variations causing artifacts, for example, abrupt changes in the perfusion (see Figures 6A,C). This is not seen as the typical spike in the perfusion signal but clearly shows up in the frequency domain. Although being an artifact in that sense, this may be useful information for clinicians. Further investigations are needed to determine the origin and its relevance to neurointensive care.

Examples of other groups that have been working on movement artifacts in LDF data show that there is no simple solution that applies to all types of LDF measurements. Humeau et al. (2009) used empirical mode decomposition in the time domain perfusion data to filter out movement artifacts in an occlusion experiment of the arm. In this case, movement artifacts could be very similar to the hyperemic response, and knowledge about the length of the hyperemia period was required beforehand. Since LDF mainly has been used on cutaneous measurements in well-defined experiments such solutions are possible. In our case, where there is no prior knowledge of the data and internal movements might be useful, other tools are needed. As a first step, thresholding the time-resolved wavelet energy works well enough. The mentioned issues may need to be further addressed before being used in real-time, but more insight into the signal and the physiology is required before an update of the signal processing is possible.

### Physiological patterns

Vasomotion (Intaglietta, 1990; Bernjak and Stefanovska, 2007; Stefanovska, 2007; Aalkjær et al., 2011) is a physiological phenomenon of high interest as it is assumed to be linked to the local regulation of tissue blood flow. In our measurements, vasomotion was found at rates of 0.9–3 cycles/min (0.015–0.05 Hz), with the most common frequency being approximately 2 cycles/min. Compared to the intervals based on the vasomotor origin, this falls into the endothelial and neurological ranges as previously published for cutaneous tissue (Stefanovska, 2007). Interestingly, a similar pattern was also found in the TLI, however, with a 180-degree phase shift (Figure 7C). To our knowledge, this is the first time this type of vasomotion-like signal is presented with LDF in the TLI. Recent investigations using high-resolution functional MRI have been able to visualize the vessel oscillations and correlate the vasomotion to the neuronal intracellular calcium signals (He et al., 2018). Hill et al. (2015) used a two-photon microscope together with transgenic mice to study variations in the microvessel diameter. Rivadulla et al. (2011) also report on the use of optical methods for the investigation of the relationship between stimulation and oscillating blood flow parameters. It is likely that it is these vessel oscillations that are displayed in the TLI signal as well. In what way vasomotion is relevant for neurointensive care is yet to be further investigated. Several researchers (Geraghty and Testai, 2017) point out the importance of not only vasospasm but also impaired cerebral autoregulation among other things, in which vasomotion plays an important role. With long-term LDF measurements of the brain microcirculation in the days following SAH or TBI, it might be possible to capture the nature of vasomotion in the injured brain.

Another interesting pattern is the short-term variations resembling artifacts in the frequency domain. They appear as a simultaneous increase in the TLI and a decrease in the perfusion in the time domain. Since having no external cause in the measurement protocol, this pattern ought to have a physiological origin. An increase in TLI means that the total amount of light reflected is higher, or the tissue is brighter. The feasibility to capture small variations in the TLI and perfusion with LDF in the human brain has previously been thoroughly investigated (Wårdell et al., 2013; Zsigmond et al., 2017). Variations were possible to distinctively separate down to 0.5 mm spatial resolution (Wårdell et al., 2016). One interesting path to investigate is if this pattern is related to CSDs, as previously described by Ayata and Lauritzen (2015) and Dreier et al. (2017) among others. In the same way as IOS has been used to detect the wavefront of a propagating CSD (Woitzik et al., 2013), the TLI ought to have the same function. Since CSDs seem to be another important mechanism in the pathophysiology after SAH and TBI, LDF with perfusion and TLI combined could be a helpful prognostic tool if CSDs can be detected by TLI and perfusion changes alone.

### Future work

The advantage of our LDF system and probe is the simultaneous recording of the perfusion and TLI at the same position. With this data as a starting point, we will collect more data and continue investigating the origin of the patterns we see in the search for information relevant to neurointensive care. For future measurements, a new probe with two channels has been designed (Mauritzon et al., 2021). This gives the possibility to measure the perfusion and TLI in two spots and a bilateral placement of the probe allows differences between the two hemispheres to be seen. More data is necessary for finding a larger set of typical patterns and linking these both to routinely measured parameters in the NICU and the patient’s status. Additional steps include correlation with other blood flow-related measurements like different MRI methods and transcranial Doppler ultrasound, and measurements during vasospasm treatment.

## Conclusion

This study demonstrates that stable cerebral perfusion and TLI signals can be recorded invasively with LDF over several days in the NICU. With a well-fixated optical probe together with signal processing, movement artifacts can be separated from physiological patterns to a satisfying extent. In total, more than 95% of the time was free from artifacts. The data showed slowly increasing trends over hours, vasomotion, and other sudden variations with physiological origin in the perfusion and TLI. These findings support the suitability of long-term LDF measurements in the NICU and, albeit clear signal patterns indicating secondary insults at this point are difficult to define, this opens possibilities for further studies and increased understanding of the human cerebral microcirculation in the injured brain. In the long run, correlations with clinical pathophysiological events after SAH and TBI can make LDF a helpful complement to established NICU monitoring techniques.

## Data availability statement

The datasets presented in this article are not readily available due to patient confidentiality and privacy reasons.

## Ethics statement

The studies involving human participants were reviewed and approved by Regionala etikprövningsnämnden i Linköping (EPN). The patient’s next-of-kin provided written informed consent for the participation in this study.

## Author contributions

KW and JH initiated and conceptualized the study. SM modified software and performed the data analysis, with input from FG. SM and KW performed the clinical measurements in close collaboration with FG and JH. SM and KW performed the overall tasks including writing and literature survey. SM performed the graphics. FG and JH contributed to the revision of clinical issues and the literature review. All authors revised the manuscript for important intellectual content.
